# Immunotherapy for tuberculosis: current strategies and future directions

**DOI:** 10.1186/s40779-025-00655-7

**Published:** 2025-10-20

**Authors:** Meng-Yuan Lyu, Hong-Li Lai, Hao-Ran Peng, Han Luo, Jian Zhou, Wan-An-Qi Ma, Chun-Ying Zhang, Hong-Xia Ruan, Yang Liu, Jie Chen, Bin-Wu Ying

**Affiliations:** 1 Department of Laboratory Medicine/Clinical Laboratory Medicine Research Center, West China Hospital, Sichuan University, Chengdu, 610041 China; 2 Sichuan Clinical Research Center for Laboratory Medicine, Chengdu, 610041 China; 3 West China School of Medicine, Sichuan University, Chengdu, 610041 China; 4 Department of Division of Thyroid and Parathyroid SurgeryWest China Hospital, Sichuan University, Chengdu, 610041 China; 5 Department of Thoracic Surgery, West China Hospital, Sichuan University, Chengdu, 610041 China

**Keywords:** Tuberculosis (TB), Cell exhaustion, Immunotherapy, Molecular mechanisms, Clinical trials

## Abstract

The worldwide dissemination of drug-resistant tuberculosis (TB) presents significant obstacles to conventional anti-TB treatment and prevention methods based on bactericidal antimicrobial drugs, greatly impeding advancements in combating this most lethal disease. With growing insights into the immunopathogenesis of TB, we are increasingly recognizing the potential of immunotherapeutic strategies aimed at targeting the host. After invading the host, *Mycobacterium tuberculosis* (*M. tuberculosis*) induces host cell exhaustion through its own molecules, such as early secretory antigen target-6 (ESAT-6) and di-O-acyl-trehalose, manifested as suppressed proliferative capacity, cytokine production, and cytotoxicity, thereby triggering the onset of TB. In response to this pathogenic mechanism, immunotherapeutic strategies, including cell therapy and immune checkpoint inhibitors, have been developed to promote cytokine production, activate immune cells to exhibit anti-TB activities such as autophagy, and restore immune homeostasis, including the balance between T helper 1 (Th1) and Th2 responses. These approaches have shown promise in restoring host immunity and demonstrating therapeutic effects against TB. However, a comprehensive evaluation of factors such as drug safety, optimal treatment duration, and others, is essential before these strategies can be integrated into routine clinical TB management. The advancement of immunotherapy has the potential to revolutionize current TB management and provide further benefits to patients. This review aims to comprehensively explore the advancements in diverse TB immunotherapeutic strategies, including efficacy, safety, and administration methods, and to explore the challenges and prospects of TB immunotherapy.

## Background

Tuberculosis (TB) remains a significant global health challenge with high rates of morbidity and mortality. It disproportionately affects low- and middle-income countries, where factors such as poverty, malnutrition, overcrowding, and limited access to healthcare contribute to its spread. Fortunately, life-saving anti-TB drugs, such as rifampicin and isoniazid, have played crucial roles in global TB control efforts [1]. These existing drugs are designed to specifically target and inhibit the growth of *Mycobacterium tuberculosis* (*M. tuberculosis*), the causative agent of TB, leading to the eventual cure of the infection. However, under drug pressure, *M. tuberculosis* adaptively evolves, exhibiting reduced growth rates, increased efflux pump activity, and increased lipid anabolism, resulting in drug resistance [2]. This traditional therapeutic strategy against *M. tuberculosis* has unavoidable limitations. Therefore, new alternative treatment strategies need to be developed.

Immunotherapies based on modulating the host immune responses have raised new hopes for combating TB. As we all know, the onset and development of TB hinges not only on the virulence and quantity of *M. tuberculosis* present but also on the immunological condition of the host [3, 4]. Upon exposure to *M. tuberculosis*, the host immune system, including both innate and adaptive immune responses, is activated to clear the pathogen. However, *M. tuberculosis* has evolved various strategies to manipulate the immune system, escape immune clearance, and further drive disease [5]. One of the key strategies is to induce cell exhaustion. This phenomenon, characterized by progressive loss of effector function, has been increasingly revealed, with its pivotal roles in the initiation and progression of TB becoming clearly established. Various immunotherapies have emerged to counteract cell exhaustion and bolster anti-*M. tuberculosis* immune responses. For example, immune checkpoint inhibitors (ICIs), which are commonly utilized in cancer therapy, have demonstrated promise in TB treatment because they block negative regulators of T-cell function and reinstate T-cell immune capacity [6, 7]. Other emerging immunotherapies, including cell therapy and various immune agents (immunoactive substances, chemical agents, vaccines, etc.), are also used to enhance immune function or decrease the level of negative regulators of immune function, promoting anti-*M. tuberculosis* activity [8–10].

Therefore, this review aims to comprehensively explore the advancements in diverse TB immunotherapeutic strategies, including efficacy, safety, and administration methods. Moreover, we delve into the challenges and prospects of TB immunotherapy, underscoring its potential to transform TB management and contribute significantly to global endeavors aimed at combating this enduring infectious disease.

## Immune evasion and cell exhaustion in TB

**Fig. 1:**
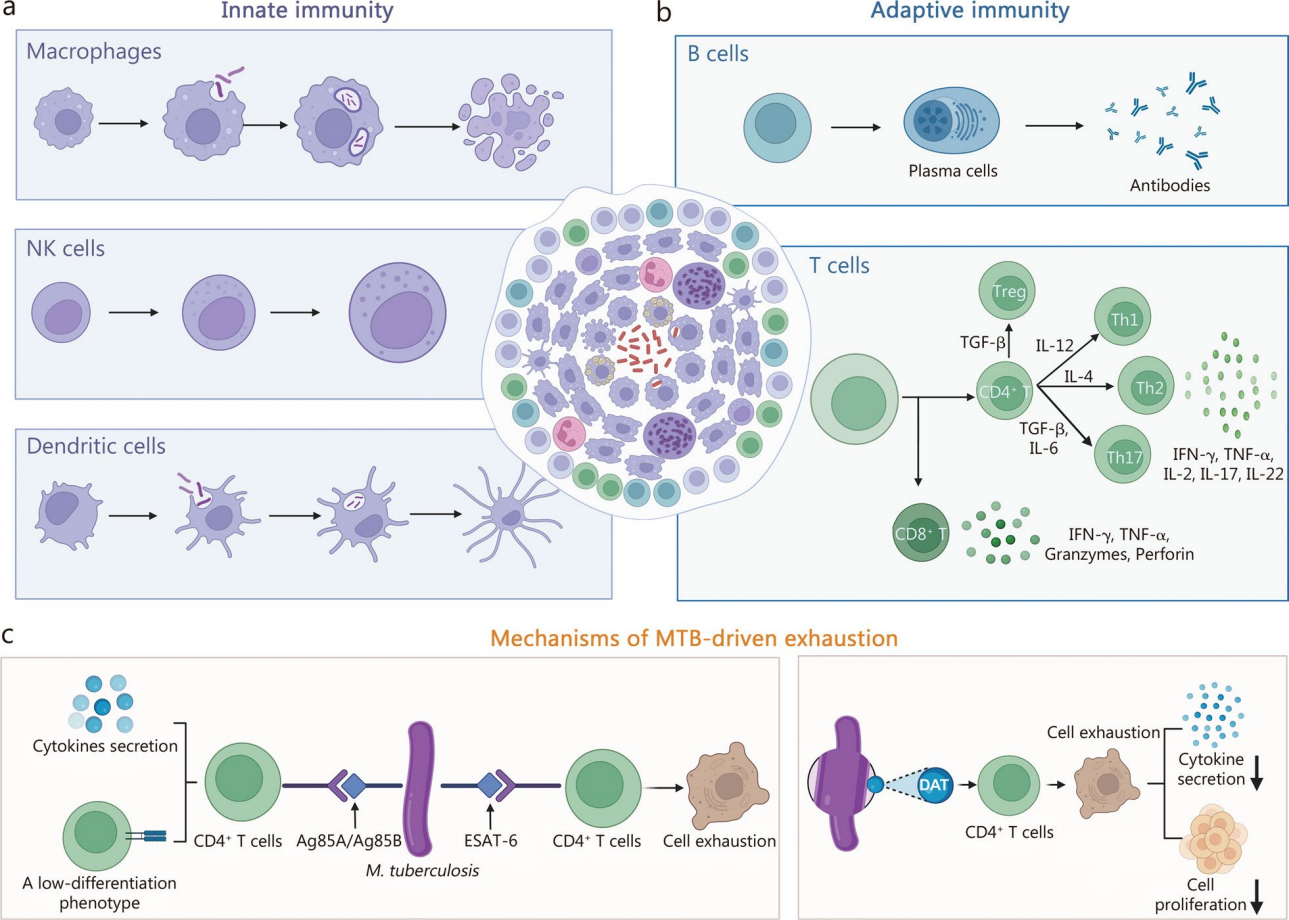
The roles of various cells in TB patients and the mechanisms of TB-associated cell exhaustion. **a** Innate immune response to *M. tuberculosis* infection. Macrophages, NK cells, and dendritic cells play major roles in recognizing and killing *M. tuberculosis* through multiple mechanisms (a subset of central mechanisms is presented). **b** Adaptive immune response to *M. tuberculosis* infection. Based on antigen presentation mediated by macrophages and dendritic cells, B-cell antibody-mediated humoral immunity and T-cell-mediated cellular immunity work together to clear *M. tuberculosis*. **c** Mechanisms underlying *M. tuberculosis*-induced host cell exhaustion. During *M. tuberculosis* infection, early antigens like Ag85A and Ag85B elicit CD4^+^ T-cell responses without inducing exhaustion, whereas persistently expressed antigens such as ESAT-6 drive T-cell dysfunction and exhaustion. Additionally, DAT from the *M. tuberculosis* cell surface may contribute to exhaustion by suppressing cytokine transcription in Th1 cells. (Created with BioRender.com). ADCC antibody-dependent cell-mediated cytotoxicity, DAT di-O-acyl-trehalose, ESAT-6 early secretory antigen target-6, IFN-γ interferon-γ, IL interleukin, *M. tuberculosis Mycobacterium tuberculosis*, NK natural killer, TB tuberculosis, TGF-β, transforming growth factor-β, TNF-α tumor necrosis factor-α

The battle between the host immune system and *M. tuberculosis* is central to understanding its mechanisms of immune evasion and cell exhaustion. This conflict begins with the host’s initiation of a complex series of immune responses following *M. tuberculosis* invasion. Upon infection, the host rapidly initiates a robust immune response. The innate immune system constitutes the first line of defense (Fig. 1a), wherein macrophages, dendritic cells (DCs), and natural killer (NK) cells recognize, engulf, and attempt to eliminate *M. tuberculosis* through mechanisms such as autophagy, apoptosis, cytotoxicity, and cytokine signaling [11–13]. These cells also orchestrate subsequent adaptive immunity by presenting antigens and releasing inflammatory mediators [14]. Adaptive immunity follows, dominated by T cell responses (Fig. 1b). CD4^+^ T helper (Th) subsets play distinct roles: Th1 cells are essential for macrophage activation via interferon-γ (IFN-γ) and tumor necrosis factor-α (TNF-α) secretion, while Th17 cells promote neutrophil recruitment and secrete cytokines such as interleukin-17 (IL-17) [15, 16]. CD8^+^ T cells lyse infected cells through cytokine release and perforin–granzyme pathways [17]. Additionally, B cells differentiate into antibody-producing plasma cells to aid in the immune effort [17]. Through coordinated innate and adaptive mechanisms, most infected individuals effectively control or clear the pathogen.

Nevertheless, *M. tuberculosis* has evolved sophisticated strategies to evade immune clearance, leading to active disease in some individuals. At the innate immunity level, the core strategy of *M. tuberculosis* is to establish a favorable replicative niche within macrophages. Through secretion of effector proteins and lipids, it inhibits phagosome maturation, neutralizes phagosomal acidity, and suppresses autophagy and apoptosis, enabling its intracellular survival and replication [18–21]. Simultaneously, *M. tuberculosis* undermines adaptive immunity by impairing T cell responses. It delays T cell activation by interfering with the antigen presentation process in DCs and can directly suppress T cell function or induce non-protective T cell responses, thereby undermining the host’s most critical line of defense [4, 12].

Based on the above mechanisms, the Directly Observed Treatment, Short-course (DOTS) strategy has been established for TB treatment [22]. This regimen involves a six-month multidrug course for drug-susceptible TB, achieving moderate success rates (around 85%) [23]. However, its efficacy is limited by poor patient compliance due to the lengthy duration, significant toxicity, and rising drug resistance [24]. Treatment of drug-resistant TB is even more challenging, requiring longer, more toxic, and costlier regimens with limited efficacy against dormant bacilli [25]. A key reason these antibiotic therapies cannot completely eradicate *M. tuberculosis* is that the pathogen has evolved even more intricate survival mechanisms [26, 27]. For instance, the *M. tuberculosis* effector mammalian cell entry 3C (Mce3C) binds and inhibits cathepsin B, which suppresses truncated BH3-interacting domain death agonist (tBID) formation and preserves receptor-interacting protein kinase 1 (RIPK1), shifting host cell death from apoptosis to necroptosis, a process that promotes bacterial persistence [26].

In parallel, *M. tuberculosis*-induced host cell exhaustion has emerged as another pivotal strategy for its survival [28]. Cell exhaustion refers to the state in which cells lose their functionality due to persistent antigen stimulation, and the phenotypic characteristics of T-cell exhaustion vary depending on the diverse natures of the pathogens [29]. Exhausted T-cells exhibit reduced proliferative capacity, diminished cytokine production, impaired cytotoxicity, and altered expression of activation markers[30]. The exhaustion state is often accompanied by the upregulation of expression of inhibitory receptors, such as programmed cell death protein-1 (PD-1), T cell immunoglobulin mucin domain-containing protein-3 (Tim-3), and cytotoxic T lymphocyte antigen-4 (CTLA-4), which further contributes to the functional impairment of the cells. During *M. tuberculosis* infection, specific antigens such as Ag85A and Ag85B are expressed in the early stages, whereas other antigens such as ESAT-6 are expressed throughout the course of infection [31]. As a result, CD4^+^ T-cells that recognize Ag85A and Ag85B exhibit a phenotype similar to that of memory cells, with low differentiation, and possess the potential to produce multiple cytokines [31, 32]. Conversely, CD4^+^ T-cells that recognize ESAT-6 undergo prolonged antigen stimulation, leading to their dysfunction and exhaustion [31, 33]. In addition to antigens, Palma-Nicolás et al. [34] discovered another mechanism of CD4^+^ T-cell exhaustion during *M. tuberculosis* infection. They reported that a lipid called di-O-acyl-trehalose (DAT) found on the *M. tuberculosis* cell surface can induce the suppression of cytokine gene transcription in CD4^+^ Th1 cells by downregulating the di-acyl glycerol-dependent activation of the mitogen-activated protein kinase (MAPK)-extracellular signal-regulated kinase (ERK)1/2 pathway. Additionally, DAT can inhibit the proliferation of antigen-stimulated T cells, which involves the decreased Th1-type cytokine transcription levels [34]. These findings shed light on the intricate mechanisms involved in T-cell exhaustion during *M. tuberculosis* infection (Fig. 1c).

## Immunotherapeutic strategies

Immunotherapy represents a diverse and rapidly evolving field, employing diverse strategies to modulate immune response. These include immunoactive substances, chemical agents, vaccines, cell therapy, and ICIs. In the following section, we provide a detailed summary of the relevant evidence for each of these approaches.

### Immunoactive substances

**Fig. 2:**
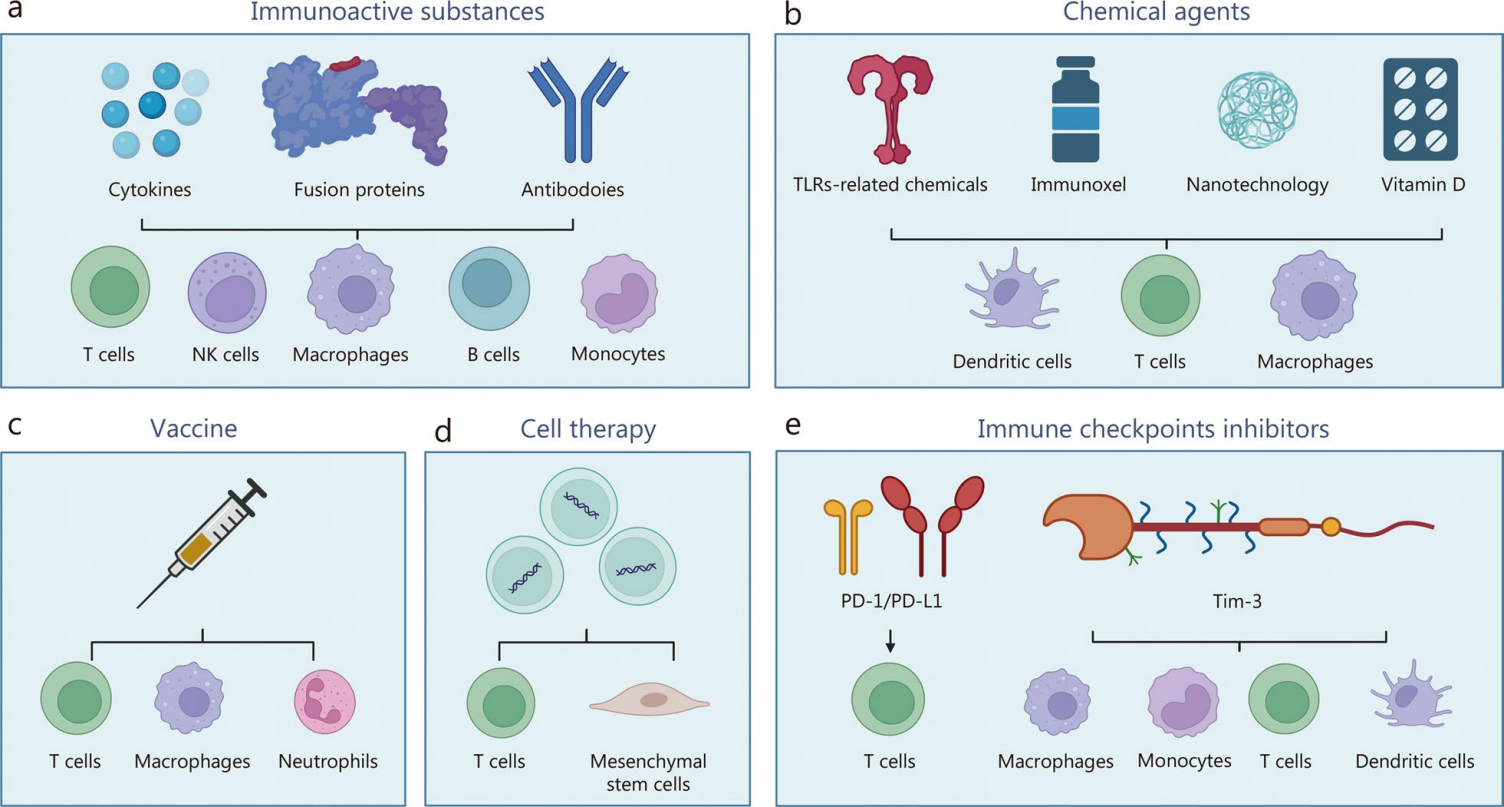
Current TB-related immunotherapy strategies and their targeted cellular objects. **a** Three key immunoactive substances (cytokines, fusion proteins, and antibodies) and their targets. **b** Four key chemical agents (TLR-related chemicals, immunoxel, nanotechnology, and vitamin D) and their targets. **c** Vaccines and their targets. **d** Cell therapy strategy and its targets. **e** Immune checkpoint inhibitors (PD-1/PD-L1 and Tim-3) and their targets. (Created with BioRender.com). NK natural killer, PD-1 programmed cell death-1, PD-L1 programmed cell death-ligand 1, TB tuberculosis, Tim-3 T cell immunoglobulin mucin domain-containing protein-3, TLR Toll-like receptor

This section provides a comprehensive description of the evidence for 3 key immunoactive substances, namely cytokines, fusion proteins, and antibodies, all of which have received significant attention for their therapeutic potential in treating TB (Fig. 2a). Additional immunoactive substances such as polypeptides, which currently have a less extensive body of research, are summarized in Table 1 [35–48] along with relevant supporting evidence.

**Table 1:** Application of other immunoactive substances in tuberculosis

Name	Subject	Clinical stage	TB subtype	Phenotype	Potential mechanism	References
Adenovirus-delivered AMP (human β-defensin 3 or cathelicidin)	Mouse	NA	Sensitive and MDR-TB	Reducing bacterial load and lung inflammation	Improving protective immune responses	[35]
GMDP	Mouse	NA	NA	Reducing bacterial load in the lungs; Selectively improving the relapse after chemotherapy	Inhibiting the O_2_ generation by phorbol myristate-induced peritoneal macrophages	[36]
L-isoleucine	Mouse	NA	Sensitive and MDR-TB	Reducing bacterial load and tissue damage	Mediating an increase in beta-defensins 3 and 4	[37]
L-isoleucine	Mouse	NA	Progressive pulmonary TB	Reducing bacterial load and pulmonary lesions	NA	[38]
IDR peptides	Mouse	NA	Sensitive and MDR-TB	Reducing bacterial load and lung inflammation	Regulating innate immunity Promoting chemokine induction and cell recruitment	[39]
Liposomally encapsulated neutrophil serine proteases (cathepsin G and neutrophil elastase)	Mouse	NA	Pulmonary TB	Prolonging the survival of infected mice; Failing to reduce bacterial load in the lungs	Regulating an unspecified inflammatory response	[40]
N-formylated peptides	Mouse	NA	NA	Reducing bacterial load in the lungs and spleen; Reducing areas of consolidation in the lungs	Mediating chemotaxis and activation of phagocytes by binding to a specific phagocyte receptor	[41]
N-formylated peptides	Mouse	NA	NA	Reducing bacterial load in the lungs and spleen	An increase in the levels of ROS in neutrophils	[42]
N-terminally formylated internal-peptide ‘f-MLLLPD’	Mouse	NA	NA	Reducing bacterial load in the lungs and spleen; Reducing pathology in the lungs	Promoting the production of ROS in stimulated neutrophils	[43]
Recombinant ESAT-6	Mouse	NA	Pulmonary TB	Reducing bacterial load in the lungs and spleen	Mediating T-cell stimulation to provide anti-TB protection; Promoting the secretion of IFN-γ and IL-4	[44]
Rhu-GM-CSF	Human	Phase II	Pulmonary TB	Promoting the conversion to negative; Inducing local skin inflammation and increased leukocyte count after each injection	Activating macrophages to inhibit or kill bacteria	[45]
The combination of recombinant IFN-γ, anti-α crystalline monoclonal IgA antibody, and IL-4 neutralizing polyclonal antibody	Mouse	NA	NA	Reducing the viable bacteria in the lungs; Preventing relapse	Improving lung granuloma area, nitric oxide, cytokine and chemokine levels	[46]
The combination of IgA 2E9 antibody and IFN-γ	Mouse	NA	MDR-TB	Reducing bacterial load in the lungs	Promoting phagocytosis and then neutrophil-induced killing of infected cells	[47]
The combination of Immunoactive drugs (thymalin and T-activin) and prednisolone	Human	NA	Chronic pulmonary TB	Interrupting bacteria isolation	Normalizing the immunological status, manifested by increased numbers and functional activity of T-cells	[48]

*AMP* antimicrobial peptides, *ESAT-6* early secretory antigen-6, *GMDP* glucosaminyl-muramyl dipeptide, *IDR* innate defense regulator, *IFN-γ* interferon-γ, *IL-4* interleukin-4, *MDR-TB* multidrug resistant, *NA* not applicable, *ROS* reactive oxygen species, *Rhu-GM-CSF* recombinant human granulocyte-macrophage colony-stimulating factor, *TB* tuberculosis

#### Cytokines

##### IL family

ILs, a family consisting of 38 members, play critical roles in immune regulation and the inflammatory response to bacterial infections. Among these members, the therapeutic efficacy of some proinflammatory factors (IL-2, IL-24, IL-12, IL-32, IL-1, IL-15, and IL-7) and antagonists of anti-inflammatory factors (IL-4 and IL-10) has been explored.

Among the proinflammatory factors, IL-2 has been extensively investigated in both animal models and clinical cohorts [49–56]. IL-2 is produced primarily by CD4^+^ T-cells and plays a crucial role in promoting the proliferation and activation of T-cells and NK cells through three signaling pathways: the Janus kinase (JAK)/Signal transducers and activators of transcription (STAT), ERK, and Phosphoinositide-3-kinase (PI3K) pathways [57, 58]. This biological function enhances the host immune response against *M. tuberculosis*. The combination of IL-2 with granulocyte–macrophage colony-stimulating factor (GM-CSF) can be beneficial for mice with multidrug resistant (MDR)-TB [49]. Mechanistically, IL-2 stimulates T-cell proliferation, and GM-CSF promotes macrophage differentiation, proliferation, and activation, further enhancing the antigen-presenting efficiency of activated macrophages [49]. In the clinic, Johnson et al. [50, 52] performed a series of studies and reported that IL-2 administration combined with routine multidrug therapy (MDT) may enhance the immune responses of acute-stage TB or MDR-TB patients, thereby benefiting these patients. The safety of this treatment has also been verified [59]. Shen et al. [53] and Liu et al. [54] reached similar conclusions regarding the therapeutic effect of IL-2 on MDR-TB patients. However, the effect of IL-2 in treating drug-susceptible pulmonary TB (PTB) patients remains controversial. Detailed results are listed in Table 2 [45, 48, 50–53, 55, 56, 60–90]. Discrepancies in the efficacy may stem from both patient heterogeneity (drug-resistant vs. drug-sensitive TB) and dosage variations across trials, necessitating rigorous investigation to delineate their respective roles.

**Table 2:** Clinical trials of TB-related immunotherapies

Name	Clinical stage	TB subtype	Sample size	Effectiveness	Safety	Administration	Dosage	References
rhuIL-2	NA	Acute or MDR TB	20	Promoting the negative conversion rate of sputum smears; Enhancing systemic responses (e.g., natural killer cell activity, cell proliferation)	No clinical deterioration or significant side effects	Injection	12.5 µg	[50]
rhuIL-2	NA	MDR TB	35	Promoting immune activation (e.g., increased CD25^+^ and CD56^+^ cells, decreased sputum bacterial load, chest radiologic improvement)	NA	Injection	12.5 µg	[51]
rhuIL-2	NA	MDR TB	14	Decreasing *IFN-γ* mRNA expression in PBMCs; Increasing IFN-γ, IL-2, cyclooxygenase-1, and hnRNP G mRNA expression at the DTH site	NA	Injection	12.5 µg	[52]
rhuIL-2	NA	MDR TB	50	Promoting the negative conversion rate of sputum smears; Chest radiologic improvement	Safe and well tolerated	Injection	500,000 U	[53]
rhuIL-2	NA	MDR TB	NA	Increasing blood CD25^+^ and CD56^+^ cells; Increasing IFN-γ and IL-2 mRNA expression at the DTH site; Reducing or clearing sputum acid-fast bacilli	NA	NA	12.5 µg	[55]
rhuIL-2	Phase II	PTB	110	No significant effect	Safe and well tolerated	Injection	225,000 U	[56]
Rhu-GM-CSF	Phase II	PTB	31	Promoting sputum conversion to negative	Inducing local skin inflammation and returning to normal in 72 h	Injection	125 mg/m^2^	[45]
Dzherelo	NA	TB/HIV coinfection	60	Regulating cytokine levels, including increased IL-2 and decreased IL-6 and TNF-α	NA	Oral	50 drops	[60]
Dzherelo	Phase II	TB/HIV coinfection	40	Increasing the absolute number of CD4^+^ T-cells	NA	Oral	50 drops	[61]
Delamanid	NA	MDR TB	23	Reducing C-reactive protein levels and inflammation	NA	NA	NA	[62]
Immunoxel	Phase III	Sensitive, MDR or XDR PTB	269	Promoting the negative conversion rate of sputum smears; Reducing TB-associated inflammation, including defervescence, and normalizing elevated leukocyte counts and ESR; Reducing anti-TB treatment-caused hepatotoxicity	No adverse effects	Sublingual	NA	[63]
Levamisole	NA	PTB	65	Promoting the negative conversion rate of sputum smears; Reducing the TB-related weight loss	Safe	Oral	100 mg	[64]
Quercetin and polyvinylpyrrolidone	NA	Destructive PTB	124	Ameliorating intoxication symptoms and respiratory symptoms	NA	Injection	0.5 g	[65]
Thymalin, T-activin, and prednisolone	NA	PTB	NA	Normalizing of the immunological status (e.g., increased count and functional activity of T-cells); Stabilizing the process with cessation of bacterial isolation	NA	NA	NA	[48]
Vitamin D and phenylbutyrate	NA	PTB	894	Ameliorating clinical TB symptoms and relevant complications; Failing to promote bacterial clearance in sputum	Reducing adverse events	Oral	Vitamin D: 5000 U; Phenylbutyrate: 2 × 500 mg	[66]
*M. vaccae*	NA	PTB	10	Comparable benefits at the clinical, bacteriological, hematological, radiological, and immunological aspects	NA	Oral and injection	1 mg	[67]
*M. vaccae*	NA	PTB	206	Promoting cure; Reducing mortality	NA	Injection	0.1 ml	[68]
*M. vaccae*	NA	PTB	22	Reducing or clearing sputum acid-fast bacilli; Reducing ESR, the rate of failed treatment or relapse; Normalizing immunological parameters; Shortening the period of chemotherapy	NA	Injection	0.1 ml	[69]
*M. vaccae*	NA	TB/HIV coinfection	1229	No significant effect on survival or bacteriological outcome	Safe and well tolerated	Injection	0.1 ml	[70]
*M. vaccae*	Phase I and II	PTB	342	Promoting the negative conversion rate of sputum smears and enhancing systemic responses (e.g., natural killer cell activity, cells proliferating); Improving X-ray manifestation; Increasing the percentage of CD3^+^ and CD4^+^ T-cells, the CD4^+^/CD8^+^ T-cell ratio, and closing of cavity; Reducing the bacteriological relapse rates	Rare and slight side effects	Injection	First time: 0.1 mg; Subsequent time: 0.5 mg	[71]
*M. vaccae*	NA	Sensitive or MDR PTB	70	Promoting the negative conversion rate of sputum smears; Chest radiologic improvement; Increasing the value of lymphocyte transformation test, CD3^+^ T-cells, CD4^+^ T-cells and CD4/CD8 T-cell ratio	NA	Injection	First time: 0.1 mg; Subsequent time: 0.5 mg	[72]
*M. vaccae*	NA	MDR TB	337	Improving the cure rate of MDR TB patients	Safe	Injection	0.1 ml	[73]
*M. vaccae*	Phase I/II	TB/HIV coinfection	120	Promoting the rate of sputum culture conversion; Chest radiologic improvement	Safe and well tolerated	Injection	NA	[74]
*M. vaccae*	NA	PTB	87	Enhancing antibody responses; Promoting the closing of cavities	NA	Injection	10^8^, 10^9^ or 2 × 10^9^ per 0.1 ml	[75]
*M. vaccae*	NA	PTB	102	Improving bacteriological success and chest X-ray (cavities and other radiological lesions); Recovering body weight; Normalizing and mean ESR	NA	Injection	0.1 ml	[76]
*M. vaccae*	NA	PTB	285	Reducing mortality	NA	Injections	NA	[77]
MIP	NA	Tuberculous pericarditis	1400	Failing to generate the significantly beneficial effect for the combined outcome of death; Reducing the incidence of pericardial constriction and hospitalization	NA	Injection	5 × 10^8^ organisms	[78]
MIP	NA	PTB	890	Promoting the conversion of sputum to negative	Safe with no adverse effects	Injection	5 × 10^8^ organisms	[79]
TB vaccine (V7)	Phase III	Sensitive, MDR or XDR PTB	152	Promoting the negative conversion rate of sputum smear; Reducing TB-associated inflammation and weight loss (e.g., defervescence, normalizing elevated leukocyte counts and ESR); Reducing treatment length; Reducing the hepatotoxicity of TB drugs	Safe	Oral	10 µg	[80]
TB vaccine (V7)	Phase II	Sensitive, retreated, drug-resistant, or HIV + PTB TB	41	Promoting clearance in sputum smears Reducing TB-associated weight loss and fever; Increasing lymphocyte counts; Shortening the period of chemotherapy	Safe	Oral	10 µg	[81]
TB vaccine (V5)	Phase II	First-diagnosed, relapsed, or MDR TB	34	Improving enlarged liver, total bilirubin, ESR, lymphocyte and leukocyte counts, and weight loss; Reducing the scoring of sputum bacillary load	No adverse effects	Oral	One tablet	[82]
BCG	NA	PTB	360	Promoting the negative conversion rate of sputum smears; Reducing the occurrence rate of MDR TB	No serious adverse reactions	Injection	0.1 mg	[83]
RUTI	Phase II	LTBI	95	NA	Safe and well tolerated	Injection	5, 25, or 50 µg	[84]
M72/AS01E	Phase II	LTBI	3573	Reducing the incidence of PTB in *M. tuberculosis*-infected adults	Increased injection-site reactions and influenza-like symptoms	Injection	2 doses	[85]
H56:IC31	Phase I/II	PTB and extrapulmonary TB	51	Significantly stimulating antigen-specific CD4^+^ T-cell proliferation	Safe	Injection	5 µg	[86]
Allogeneic Vγ9Vδ2 T-cell therapy	NA	MDR TB	8	Showing clinical efficacy in multiple aspects, including promoting the repair of pulmonary lesions and alleviating *M. tuberculosis* load in vivo	Safe	Injection	10^8^ cells	[87]
Autologous MSC therapy	NA	MDR or XDR PTB	27	Enhancing the therapy efficiency	NA	NA	NA	[88]
Autologous MSC therapy	NA	MDR or XDR PTB	30	NA	Safe	Injection	10^6^ cells/kg	[89]
CIK	NA	MDR TB	18	Promoting the negative conversion rate of sputum smears; Chest radiologic improvement; Relieving clinical symptoms such as cough	Safe	Intravenous infusion	10^9^ cells	[90]

*BCG Bacillus Calmette-Guérin*, *CIK* cytokine-induced killer, *DTH* delayed-type hypersensitivity, *ESR* erythrocyte sedimentation rate, *HIV* human immunodeficiency virus, *hnRNP* heterogeneous nuclear ribonucleoprotein, *IFN-γ* interferon γ, *IL-6* interleukin-6, *LTBI* latent TB infection, *MDR* multidrug resistant, *MIP Mycobacterium indicus pranii*, *MSC* mesenchymal stem cell, *M. tuberculosis Mycobacterium tuberculosis*, *M. vaccae Mycobacterium vaccae*, *NA* not applicable, *PBMCs* peripheral blood mononuclear cells, *PTB* pulmonary tuberculosis, *rhuIL-2* recombinant human interleukin-2, *TB* tuberculosis, *TNF-α* tumor necrosis factor-α, *XDR* extensively drug-resistant

For the rest of proinflammatory factors, their therapeutic efficacies have been confirmed only in animal models. IL-24 was initially recognized as a tumor suppressor and was subsequently found to regulate multiple immune and nonimmune cell functions, mainly through the JAK/STAT signaling pathway [91, 92]. For example, it can interact with the IL-20 receptor and subsequently activate the JAK/STAT3 pathway to promote the secretion of the proinflammatory mediators IL-8, prostaglandin E2 (PGE2) and matrix metallopeptidase-1 (MMP-1) [93]. It can also impede B-cell maturation into plasma cells and has different regulatory effects on the activation of effector T-cells and the production of IFN-γ [91, 94]. In TB patients, IL-24 has been shown to activate CD8^+^ T-cells and promote the production of IFN-γ, exerting anti-TB effects and conferring a protective effect against *M. tuberculosis* infection in mice [95, 96]. Likewise, IL-12primarily functions on lymphocytes, binding to the IL-12 receptors in T-cells and NK cells, activating the JAK2/ tyrosine kinase 2 (TYK2) /STAT4 pathway and promoting the secretion of IFN-γ, which aids in the clearance of *M. tuberculosis* [97–100]. Nolt et al. [101] reported that IL-12 treatment can improve the survival of only CD4^+^ T-cell-deficient mice but not wild-type mice. They reported that this treatment can cause serious adverse effects (e.g., hepatic dysfunction or death), which may limit its application in the clinic.

IL-1 and IL-32 act primarily on macrophages and enhance bacterial killing by inducing several processes, including apoptosis and pyroptosis [11, 102–104]. IL-15 and IL-7 can activate both T-cells and macrophages to reduce the *M. tuberculosis* burden and increase the survival of infected mice [105].

Two anti-inflammatory factors, IL-10 and IL-4, exhibit potent immunosuppressive roles in the host response to *M. tuberculosis*. Reduced levels of either cytokine have been associated with decreased bacterial loads and conferred protection in murine infection models [106–108]. Mechanistically, IL-10 interferes with the apoptosis of macrophages by reducing the production of TNF-α. It also attenuates the Th1 immune responses by inhibiting IL-12 production and major histocompatibility complex (MHC) class II expression [109, 110]. Additionally, IL-10 can disrupt phagosomal maturation in a STAT3-dependent manner [111, 112]. Through these mechanisms, IL-10 promotes immune evasion of *M. tuberculosis* and facilitates its intracellular growth [113]. Similarly, IL-4 supports in *M. tuberculosis* replication by counteracting IgA/IFN-γ-mediated macrophage activation and inhibiting nitric oxide (NO) production in macrophages [108, 114].

Collectively, the existing preclinical and limited clinical evidence indicates promising potential for supplementation with pro-inflammatory factors or antagonists of anti-inflammatory factors as immunotherapeutic strategies against TB. However, clinical translation remains challenged by the short half-lives of these biologic agents [115]. Strategies such as molecular engineering for improved stability, novel delivery systems, and alternative formulation designs may help extend therapeutic duration and reduce treatment costs. Furthermore, comprehensive large-scale clinical trials remain essential to establish the efficacy and safety profiles of these interventions.

##### Transfer factor (TF) family

TFs are low-molecular-weight dialyzable lymphocyte extracts known for their immune-stimulating properties [116]. TFs primarily exert their effects on cell-mediated immunity by promoting the synthesis of migration inhibitory factor and IFN-γ [117]. Additionally, TFs can inhibit the activation of nuclear factor κ- (NF-κ) light-chain-enhancer of activated B-cells, leading to reductions in TNF-α and IL-4 levels [116, 118]. Early clinical studies in the 1970s reported improvements in the clinical conditions of TB patients following TF treatment [119]. However, there was a gap in TF research until 2004, when an animal experiment was conducted. In TB-related mouse models, TFs have demonstrated dose-dependent beneficial effects [118]. Additionally, TFs show a synergistic effect when used in combination with conventional chemotherapy, leading to significantly faster elimination of *M. tuberculosis* from the lungs than does chemotherapy alone [118]. These observations are based primarily on animal models and isolated treatments in TB patients. Further mechanistic studies are required to elucidate the underlying molecular pathways, followed by well-designed clinical trials to establish their efficacy and safety in TB patients.

#### Fusion proteins

Owing to advances in proteomic technologies, fusion proteins comprising different proteins, domains, or peptides have been developed to attain target proteins with improved properties [120]. These fusion proteins overcome the defects of the native proteins and acquire multiple advantageous properties [121, 122].

GM-CSF is a hematopoietic growth factor that plays a crucial role in the production, proliferation, maturation, and activation of myeloid cells such as monocytes and macrophages [123–125]. In TB patients, GM-CSF has been shown to increase the ability of macrophages to suppress the growth of *M. tuberculosis*, and the administration of this cytokine in vitro aids in controlling infection in cells [126, 127]. These findings indicate the tremendous value of GM-CSF as an immunotherapeutic agent against *M. tuberculosis*. However, limitations in the application of GM-CSF in anti-TB treatment arise from its physicochemical properties, such as a short half-life and poor targeting ability. To address the aforementioned limitations, Chuang et al. [128] engineered a fusion protein, albumin-fused GM-CSF (albGM-CSF). This fusion protein has a prolonged half-life and improved targeting ability. Functionally, albGM-CSF promotes the proliferation of DCs, inducing the activation of naïve T-cells and an effective immune response [128]. It can also enhance lipopolysaccharide (LPS)-induced IL-1β secretion from DCs and macrophages by activating NF-κB signaling [128]. IL-1β has been demonstrated to control *M. tuberculosis* growth during chronic infection [129]. In terms of treatment efficiency, mice treated with albGM-CSF exhibited higher IL-1β levels compared to those receiving conventional GM-CSF, indicating a stronger capacity to control *M. tuberculosis* infection. Importantly, the intravenous or subcutaneous route is considered optimal for enhancing the therapeutic efficacy of albGM-CSF [128]. Clinically, Pedral-Sampaio et al. [45] have reported that use of GM-SCF can promote sputum conversion to negative with causing local skin inflammation, indicating its application potential.

Other fusion proteins, including Rv2882c-Rv2005c and DABIL-4, have also been reported to exert anti-TB effects through various pathways, such as generating persistent and antigen-specific multifunctional CD4^+^ T-cells; promoting the production of some anti-TB cytokines (e.g., TNF-α, IFN-γ and IL-2); reducing the number of polymorphonuclear myeloid-derived suppressor cells (MDSCs) and monocytic MDSCs; and increasing the number of IFN-γ ^+^ T-cells [130, 131].

The development of fusion proteins like albGM-CSF offers a promising avenue for TB immunotherapy. Future research should prioritize optimizing their design for enhanced stability and delivery, validating efficacy and safety in clinical trials, and exploring novel fusion combinations targeting host-directed pathways. Interdisciplinary efforts will be essential to translate these innovations into practical TB treatments.

#### Antibodies

Given the intracellular parasitic nature of *M. tuberculosis*, cellular immune responses have been the focus of TB research. Recently, accumulating evidence has revealed that there is a significant correlation between low levels of *M. tuberculosis*-specific antibodies and increased susceptibility to TB in patients [132–134]. In in vivo experiments, administration of intravenous immunoglobulin has been reported to provide protection against PTB in mice, particularly when intact Fc oligosaccharides are present [135]. *M. tuberculosis*-specific antibodies can exhibit a range of beneficial effects in various ways, including regulating inflammation, activating complement, promoting phagosome maturation, increasing macrophage Ca^2+^ signaling and intracellular killing, and facilitating the clearance of immunomodulatory antigens (e.g., lipoarabinomannan) [136–138]. These findings emphasize the importance of humoral immunity in TB patients, leading to increased attention being given to antibody-based anti-TB therapeutic strategies. IgG antibodies targeting arabinomannan (9D8 antibody) [139], heparin-binding hemaglutinin (4057 antibody) [140], and arabinomannan in lipoarabinomannan (SMITH14 antibody) [141] can protect infected mice by reducing bacterial load, controlling TB dissemination, and prolonging survival. Both TBA61 [142, 143] and 2E9 [144] IgA antibodies are designed to target α-crystallin, which conveys protective effects (e.g., reduced bacterial load and prolonged survival) by inducing a proinflammatory cellular response and promoting the apoptosis of infected macrophages.

The accumulating evidence supporting the protective role of humoral immunity against tuberculosis underscores the promise of antibody-based therapeutic strategies. Future efforts should prioritize the identification of novel antigen targets, optimization of antibody engineering for enhanced effector function and half-life, and validation of efficacy in well-designed clinical trials. Combining antibody therapy with existing antibiotics or host-directed therapeutics may represent a powerful approach for improving TB treatment outcomes, particularly in drug-resistant cases.

### Chemical agents

Chemical agents are widely used as adjunctive medications during anti-TB therapy [10]. These agents exert their functions through various mechanisms, such as inhibiting the antibacterial activity of phagocytes, increasing Th1-associated cytokine levels, decreasing Th2 expression, and suppressing IL-4 secretion [145]. The anti-TB effects of Toll-like receptor (TLR)-related chemicals, immunoxel, nanotechnology, and vitamin D are summarized in this section (Fig. 2b). The relevant evidence related to other agents is shown in Table 3 [62, 64–66, 145–160].

**Table 3:** Application of other chemical agents in tuberculosis models

Name	Subject	Clinical stage	TB subtype	Phenotype	Potential mechanism	References
Ag85AB-CpG-DDA	Mouse	NA	NA	Reducing bacterial burden in the lungs and spleen	Promoting the generation of type-I cytokines and CD44-positive T-cells; Inhibiting the secretion of IL-4	[146]
Allicin	Mouse	NA	Sensitive, MDR- and XDR-TB	Reducing bacterial burden; Reversing the immune-dampening effects of anti-TB drugs	Mediating the generation of pro-inflammatory cytokines in macrophages and the protective Th1 response	[147]
All-trans retinoic acid, 1,25(OH)_2_-vitamin D_3_ and α-galactosylceramide	Mouse	NA	Pulmonary TB	Reducing bacterial burden	Reduced the accumulation of immature myeloid cells in the lungs; Increasing the level of TNF-α	[148]
Bacterial ghosts	Mouse	NA	TB	Promoting the killing of bacteria	Mediating macrophage activation by promoting the production of nitric oxide; Enhancing the influx of innate and adaptive effector immune cells and increasing the production of key cytokines in the lungs	[149]
Bergenin	Mouse	NA	MDR- and XDR-TB	Reducing bacterial burden; Reducing immune impairment and treatment duration	Mediating Th1- and Th17 cell-induced protective immune responses; Promoting long-lasting, antigen-specific central memory T-cell responses	[150]
Delamanid	Human	NA	MDR-TB	Reducing the level of C-reaction protein and inflammation	Inhibiting CXCL10 expression by regulating JAK2/STAT1 signaling	[62]
Denileukin diftitox	Mouse	NA	Acute TB	Improving the effectiveness of standard anti-TB treatment; Reducing bacterial burden	Reducing regulatory T-cells and myeloid-derived suppressor cells	[151]
Heat-killed *Caulobacter crescentus*	Mouse	NA	NA	Reducing disseminated *M. tuberculosis* in the lungs, liver, and spleen	Mediating the rapid production of specific cytokines	[152]
Levamisole	Human	NA	Pulmonary TB	Promoting the negative conversion rate of sputum smear; Reducing TB-related weight loss	Promoting the shift from Th2-type cytokine response to Th1-type cytokine response; Reducing the level of TNF or the susceptibility of normal tissue to TNF by immunomodulatory action	[64]
Luteolin	Mouse	NA	NA	Reducing anti-TB treatment duration and preventing relapse	Enhancing long-term anti-TB immunity by promoting central memory T-cell responses; Enhancing the activity of natural killer cells and natural killer T-cells	[153]
NiuBeiXiaoHe	Mouse	NA	NA	Reducing bacterial load and histopathological lesions	Inhibiting the supply of necessary ingredients during bacterial growth and proliferation	[154]
Quercetin and polyvinylpyrrolidone	Human	NA	Destructive pulmonary TB	Ameliorating intoxication symptoms and respiratory symptoms	Increasing the level of IL-4 and reducing the levels of IL-1β and TNF-α in serum	[65]
Soluble betaglycan and niflumic acid	Mouse	NA	Pulmonary TB	Reducing pulmonary inflammation, fibrosis, and bacillary load	Increasing Th1, but decreasing Th2 cytokines; Promoting the expression of iNOS	[145]
Sphingosine 1-phosphate	Mouse	NA	Primary and acute TB	Reducing infected cells and bacterial load in the lungs and spleen during primary infection, but not during acute infection; Reducing histopathology	Regulating the activity of multiple cells, including T-cells and macrophages	[155]
Suplatast tosylate and D4476	Mouse	NA	TB	Promoting *M. tuberculosis* clearance	Mediating central memory T-cell responses; Enhancing Th1 responses	[156]
Vitamin D and phenylbutyrate	Human	NA	Pulmonary TB	Ameliorating clinical TB symptoms and relevant complications; Failing to promote the clearance of bacteria in the sputum	Promoting macrophage-mediated *M. tuberculosis* killing by inducing the antimicrobial peptide LL-37 and autophagy; Mediating LL-37 in different cell types and autophagy in macrophages; Mediating innate mucosal immunity and exerting anti-inflammatory functions such as the inhibition of dendritic cell maturation, Th1/Th17 cell proliferation, and cytokine production	[66]
Withaferin A	Mouse	NA	NA	Inhibiting intracellular drug-sensitive and -resistant *M. tuberculosis*; Reducing TB recurrence	Inducing the differentiation of host macrophages to defensive M1 polarization; Enhancing Th1 and Th17 immune responses; Inducing the generation of T-cell memory cells by activating STAT signaling	[157]
Zoledronate and IL-2	Macaque	NA	MDR-TB	Increasing *M. tuberculosis* clearance in sputum and conversion rate; Reducing bacterial load and pathology/lesions	Expanding Vγ2Vδ2 T-cells and promoting the generation of anti-TB cytokines in Vγ2Vδ2 T-effector cells; Increasing the abundance of circulating CD4^+^ Th1 and CD8^+^ Th1-like effector cells	[158]
[6]-Gingerol	Mouse	NA	MDR- and XDR-TB	Reducing bacteria growth in the lungs, spleen, and liver	Promoting pro-inflammatory cytokine expression; Enhancing Th1/Th17 responses	[159]

*Ag85AB-CpG-DDA* unmethylated CpG motif-containing oligonucleotide and dimethyldioctadecylammonium bromide, *CXCL10* C-X-C chemokine ligand 10, *IL-4* interleukin-4, iNOS inducible nitric oxide synthase, *JAK* Janus kinase, LL-37 cathelicidin antimicrobial peptide, *MDR* multidrug resistant, *M. tuberculosis Mycobacterium tuberculosis*, *NA* not applicable, *STAT* signal transducers and activators of transcription, *TB* tuberculosis, *Th T* helper cell, *TNF-α* tumor necrosis factor-α, *XDR* extensively drug resistant

#### TLRs-related chemicals

TLRs, a type of pattern recognition receptor (PRR), recognize a wide range of microbial ligands and pathogen-associated molecular patterns (PAMPs), thereby stimulating innate immune cells to enhance the expression of cytokines, chemokines, and antimicrobial prorteins [161–163]. Upon stimulation, TLRs activate downstream signaling pathways such as those related to mitogen-activated protein kinase (MAPK) and NF-κB, thereby regulating an effective immune response against TB [162].

TLR-4 is a prominent player in the innate immune system and can form a complex with myeloid differentiation factor 2 (MD2), which is activated upon interaction with LPS [164]. This complex can induce the production of proinflammatory cytokines and type 1 interferons through MyD88-dependent and MyD88-independent pathways, respectively, thereby regulating the immune response during infection [165]. In the context of TB, TLR-4 signaling exhibits a dual role in host–pathogen interactions. On one hand, *M. tuberculosis* can upregulate specific microRNAs in a TLR-4-dependent manner to suppress NF-κB activation and inflammatory cytokine production, thereby dampening host immunity and promoting its intracellular survival [166, 167]. On the other hand, TLR-4 also mediates protective responses through inducing autophagy, a well-known mechanism for controlling intracellular survival of *M. tuberculosis* [168]. Given its complex involvement in TB immunity, TLR-4 has emerged as a promising target for anti-TB therapy [168–170]. For example, the combined activation of TLR-4 and CD40 triggers DCs to secrete immune-stimulating cytokines such as IL-12, IL-6, and TNF-α, which effectively activate T-cells, a cell population critically involved in the control of TB [168, 171]. This combination therapy also influences other TB-associated processes, including promoting autophagy, increasing NO release, and facilitating the production of IFN-γ and IL-17, collectively strengthening TB control [168]. Similarly, the combination of TLR-4 and NOD-2 enhances DCs function by promoting the release of IL-6, IL-12, NO, inducing autophagy, and facilitating migration to lymph nodes. These responses collectively restrict the intracellular survival of *M. tuberculosis* and promote T-cell activation, thereby modulating the anti-TB immune response [170]. Moreover, certain compounds, such as P-MAPA (an immunomodulatory agent of TLR-2 and TLR-4) and specific TLR-4 agonists, play an anti-TB role by promoting TLR-*M. tuberculosis*-derived component interactions, Th1-type cytokine responses, the maturation of DCs and macrophages, and thus the production of IFN-γ [172–174]. In addition to TLR-4 and its relevant compounds, its ligands are also considered to have therapeutic effects. TLR-4 ligand (TLR-4L) administered in vivo significantly enhances expression of the CD69^high^/CD44^high^/CD62L^low^ phenotype on CD4^+^ and CD8^+^ T-cells, consequently promoting the generation of effector memory T-cells. These memory T-cells exhibit rapid responses, including recognizing and targeting infected cells, releasing some cytotoxic molecules to eliminate bacteria, and preventing further spread [170]. Clinically, the combined application of TLR-4L, NOD-2L, and anti-TB drugs may reduce the necessary drug dose and treatment duration, demonstrating the beneficial effects of such strategies [170].

TLR-2 is another important member that contributes to TB-related immune activities. Many *M. tuberculosis* components can interact with TLR-2; for example, the 19 kD lipoprotein is a ligand of TLR-2. *M. tuberculosis* utilizes its TLR-2 ligands to impair macrophage functions by suppressing antigen presentation through downregulation of MHC class II expression, blocking the response to IFN-γ, and inhibiting autophagy [137, 175, 176]. These mechanisms contribute to the persistent survival of *M. tuberculosis*. TLR-2 agonists have been reported to have a significant effect on the intracellular survival of *M. tuberculosis*. Some of them, such as Pam2Cys and LpqH, have been reported to significantly affect the intracellular survival of *M. tuberculosis* by increasing the production of IFN-γ, increasing the expression of memory markers (CD44^high^/CD62L^high^) on CD4^+^ T-cells, promoting a Th1 immune response, and activating apoptosis and autophagy [177–180]. These processes ultimately reduce the *M. tuberculosis* load.

Future efforts should focus on developing combination therapies that integrate TLR agonists with standard antibiotics to enhance treatment efficacy against TB. Key priorities include optimizing agonist selection, delivery systems, and dosing schedules to maximize immune activation while minimizing adverse effects. Translational studies and clinical trials are essential to validate these strategies for reducing treatment duration and combating drug-resistant TB.

#### Immunoxel

Immunoxel (Dzherelo) is a multiherbal oral immunomodulator recommended by Ukrainian health authorities as an adjunct treatment for TB [181]. Dzherelo can promote the production of IFN and restore cellular and humoral immunity, thus eliminating infections. Nikolaeva et al. [60] reported that the combination of Dzherelo, Anemin (a phytoconcentrate used in Ukraine), and standard anti-TB therapy (ATT) provides greater benefits to patients with active PTB than does chemotherapy alone. Dzherelo has also been found to be beneficial for TB/human immunodeficiency virus (HIV)-coinfected patients, potentially because of its ability to increase the counts of CD3^+^ and CD4^+^ lymphocytes and normalize lymphocyte homeostasis [61]. Other forms of immunoxel (honey lozenges, etc.) and their therapeutic effects have also been reported in TB patients [63]. Regarding safety, no obvious side effects have been reported.

Future research on Immunoxel (Dzherelo) should include well-designed clinical trials to validate its adjunctive benefits, especially in TB/HIV coinfection and drug-resistant TB. Further investigation into its active ingredients, mechanisms, and drug interactions is also needed. Standardization of its formulations and treatment protocols will be crucial to support wider therapeutic application.

#### Nanotechnology

Nanotechnology, which involves the engineering and manufacturing of materials at the nanoscale, holds great potential in clinical diagnosis and treatment because of its unique advantages, including prolonged blood circulation, strong targeting abilities, and efficient delivery [182–186]. Recently, renewed interest has been sparked in the potential of nanotechnology for anti-TB immunotherapy.

**Fig. 3:**
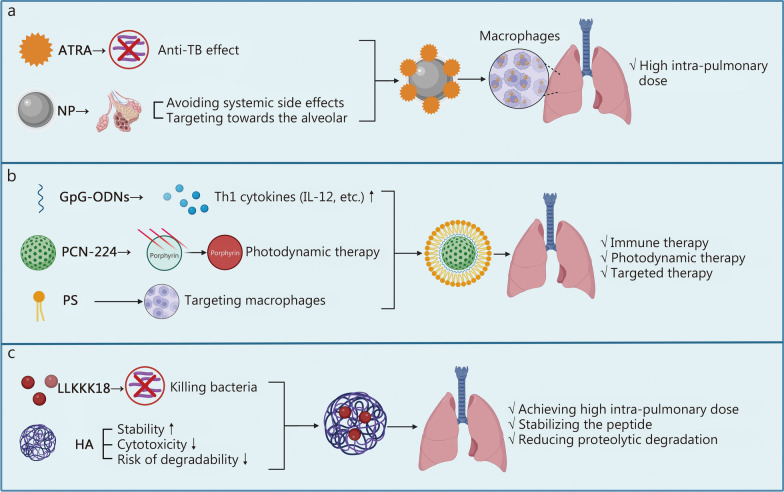
Characteristics of emerging nanotechnology in the field of antituberculosis treatment. **a** ATRA-loaded nanoparticles. This system enables the aerosolized delivery of ATRA directly to the lungs, overcoming its pharmacokinetic limitations to promote autophagy and modulate immune cells in infected macrophages.** b** PCN-CpG@PS nanocomposites. A biomimetic core–shell nanostructure that synergizes photodynamic therapy (via porphyrin in PCN-224), immune activation (via TLR-stimulating CpG ODNs), and precise macrophage targeting (via the PS coating). **c** LLKKK18-loaded nanogels. HA nanogels encapsulating the antimicrobial peptide LLKKK18, enhancing its stability, macrophage uptake, and bactericidal efficacy against intracellular *M. tuberculosis*. (Created with BioRender.com). ATRA all-trans retinoic acid, CpG-ODNs CpG oligodeoxynucleotides, HA hyaluronic acid, IL-12 interleukin-12, NP nanoparticle, PS phosphatidylserine, TB tuberculosis, Th1 T helper 1

One example is the use of nanoparticles (NPs) to deliver all-trans retinoic acid (ATRA) (an active metabolite of vitamin A, also known as tretinoin) (Fig. 3a). ATRA plays diverse roles in anti-TB immune activity, such as promoting autophagy in infected macrophages, inducing the maturation and exhaustion of MDSCs, and inhibiting T-cell proliferation [187, 188]. However, ATRA administration faces challenges such as poor solubility, a short plasma half-life, an inability to reach therapeutic concentrations in target tissues, and potential off-target systemic toxicity [189, 190]. To overcome these obstacles, Bahlool et al. [191] developed a host-directed therapy (HDT) by loading ATRA into NPs. This approach enables the aerosolized delivery of ATRA to the lungs, achieving high intrapulmonary doses and facilitating uptake by infected cells. Improved drug delivery efficiency could improve patient prognosis and reduce the risk of drug resistance.

Another example is the construction of a nanocomposite called PCN-CpG@PS (a name derived from its components) (Fig. 3b), which consists of CpG oligodeoxynucleotides (CpG ODNs), PCN-224, and phosphatidylserine (PS) [192]. CpG ODNs are synthetic oligodeoxyribonucleotide sequences that can induce the production of Th1 cytokines (e.g., IL-12) by stimulating endolysosome-derived TLRs, thereby promoting a robust Th1 immune response. However, the application of CpG ODNs is limited because of their simple degradation and difficulty in crossing cell membranes due to their negative charge [193]. PCN-224 overcomes these limitations by providing a high drug loading capacity. Furthermore, a photosensitizer molecule in PCN-224 called porphyrin can perform photodynamic therapy (PDT) simultaneously with the delivery of CpG ODNs, enhancing the treatment effect [192]. PS enables precise targeting of macrophages. Therefore, this nanocomposite enables immune, photodynamic, and targeted therapy. The effectiveness and safety of these nanocomposites have been fully evaluated in vitro [192].

LLKKK18-loaded nanogels represent another promising candidate for anti-TB strategies (Fig. 3c) [194]. LLKKK18 is an antimicrobial peptide (AMP) with effective bactericidal properties, which are further enhanced when loaded into hyaluronic acid (HA) nanogels [195]. LLKKK18-loaded nanogels exhibit high stability, low cytotoxicity, and low degradability risk [194]. Importantly, the efficient internalization of nanogels by macrophages enhances the targeting ability of AMP, effectively reducing the *M. tuberculosis* load in infected macrophages [194]. Additionally, other agents developed using nanotechnology, such as poly(lactic-co-glycolic acid) (PLGA) nanoparticles, imidazoquinoline-ligated nanogels, and curcumin nanoparticles, have also demonstrated excellent anti-TB effects [196–198].

Future development of nanotechnology-based TB immunotherapies should focus on optimizing nanoparticle design for improved targeting, controlled release, and enhanced biocompatibility. Preclinical studies using advanced animal models are essential to evaluate efficacy, dosage, and synergy with existing antibiotics. Clinical translation of these systems may enable more effective host-directed therapies, shorten treatment duration, and improve outcomes against drug-resistant TB.

#### Vitamin D

Vitamin D is an essential fat-soluble vitamin for human health. In addition to its role in regulating calcium and phosphorus metabolism, its immunomodulatory properties have attracted increasing interest related to various diseases [199, 200]. In TB patients, decreased levels of vitamin D are common, and such a deficiency is significantly related to an increased risk of both TB and MDR-TB [201, 202]. Consequently, restoring vitamin D levels is considered a potential adjunct therapy for TB. As for the safety of this therapy, no serious adverse events have been reported. However, the conclusions of published clinical trials regarding the efficacy have been inconsistent. Some studies have reported that vitamin D supplementation can improve clinical symptoms, increase the rate of negative sputum conversion, shorten the duration of sputum conversion, and reduce mortality [66, 203–206]. However, some other studies failed to find a beneficial effect of vitamin D supplementation in TB patients [207–209]. Mechanistically, vitamin D can induce the production of the antimicrobial peptide LL-37, the production of nitrogen and oxygen reactants, and autophagy to enhance the macrophage-mediated killing of *M. tuberculosis* [210, 211]. In addition, vitamin D promotes the expansion of regulatory T-cells and limits Th1-mediated inflammatory responses to prevent excessive inflammation and tissue damage [212]. These contradictory results may be attributed to differences in vitamin D supplementation protocols (dosage, duration, intervals, etc.) and variations among patients (genetic backgrounds, geographical locations, etc.). Additional research is needed to elucidate the precise influence of these factors on the therapeutic efficacy of vitamin D in TB patients and to establish the optimal parameters for its therapeutic utilization.

### Vaccines

Due to their advantages, such as simple preparation and minimal side effects, vaccines have become a primary method for disease prevention. In recent years, new roles for vaccines have been explored, including their potential as therapeutic options [77, 83]. Existing vaccines can be categorized into 5 main types: inactivated vaccines, live attenuated vaccines, adjuvanted subunit vaccines, viral vector vaccines, and nucleic acid vaccines. Here, we summarize and discuss the relevant evidence for each type of vaccine related to anti-TB treatment. Figure 2c illustrates the targeted cellular objects of these vaccines. Information regarding additional vaccines is provided in Table 4 [213–215].

**Table 4:** Application of other vaccines for tuberculosis

Name	Subject	Clinical stage	TB subtype	Phenotype	Potential mechanism	References
Alternating MHC-class II restricted peptide vaccination	Mouse	NA	NA	NA	Enhancing central and effector CD8 memory; Inducing strong cytotoxic T lymphocyte responses	[213]
Recombinant *Mycobacterium smegmatis*	Mouse	NA	Persistent TB	Reducing bacterial load and pathological tissue damage	Promoting the generation of IFN-γ; Inhibiting the Th1-type immune response	[214]
Recombinant *Mycobacterium smegmatis*	Mouse	NA	NA	NA	Increasing the levels of serum IFN-γ, IL-12, and IgG2a; Inducing the proliferation of purified protein derivative antigen-specific splenocytes; Mediating the specific Th1 immune response	[215]

*MHC* major histocompatibility complex, *NA* not applicable, *TB* tuberculosis, *IFN-γ* interferon-γ, *Th1* T helper 1, *IL-12* interleukin-12

#### Inactivated vaccines

Inactivated vaccines represent a classical yet continually evolving immunization strategy against TB. These formulations are based on whole-cell or fragmented mycobacterial pathogens that have been inactivated to eliminate pathogenicity while retaining immunogenicity. There are 4 key vaccines in this category: the *M. vaccae* vaccine, the *M. indicus pranii* (MIP) vaccine, DAR-901, and RUTI. Among these vaccines, the *M. vaccae* vaccine has received approval for adjuvant therapy in patients with active TB, whereas the others are currently in clinical trials [216].

##### M. vaccae vaccine

*M. vaccae* is a nonpathogenic species of the Mycobacteriaceae family belonging to the same genus as *M. tuberculosis* [217]. The *M. vaccae* vaccine is capable of stimulating T-cells to produce more cytokines (e.g., IL-12 and TNF-α), enhancing IFN-γ responses, inducing the release of proinflammatory factors via MyD88-dependent TLR signaling, and promoting PI3K/protein kinase B (Akt) signaling pathway-dependent apoptosis; resulting in a relatively strong immunotherapeutic effect [217–219]. It has been applied as an immunotherapeutic adjunct for the treatment of TB [220–222], with clinical trials generally indicating an acceptable safety profile, though mild adverse effects have been reported in a subset of patients [71, 80, 81]. Studies across diverse patient populations have demonstrated its benefits in promoting negative sputum conversion, improving cure rates, attenuating symptoms, and reducing anti-TB drug-induced hepatotoxicity in pulmonary TB, as well as showing potential in MDR-TB [68, 69, 72–76]. Nevertheless, its efficacy remains controversial in certain contexts, particularly concerning dormant TB [223] and TB/HIV coinfection [70]. Gröschel et al. [224] also failed to reveal a significant benefit of this vaccine by conducting a systematic meta-analysis, suggesting that underlying factors may contribute to such inconsistencies. One possible explanation lies in the inherent heterogeneity of the vaccine formulations. Supporting this notion, Rodríguez-Güell et al. [225] demonstrated in a mouse model that the rough colonial variant of *M. vaccae* can induce higher production of IFN-γ and IL-12 compared to the smooth variant. This indicates that morphological and functional differences between bacterial variants may directly influence immunogenicity, highlighting the importance of standardizing morphological characteristics during vaccine preparation to ensure consistent efficacy.

##### MIP vaccines

The MIP vaccine is derived from killed *M. indicus pranii*, a nonpathogenic mycobacterial strain. Mechanistically, this vaccine triggers autophagy and the TLR signaling pathway, which activates innate immunity, stimulates the T-cell response, and enhances the ability of macrophages to clear *M. tuberculosis* [226]. Phase I to III clinical trials have demonstrated the therapeutic potential of this vaccine [227]. Research has indicated that its use can improve the sputum-negativity rate and shorten the conversion time [79]. Nonetheless, this efficacy appears to be limited in patients with tuberculous pericarditis, with reports of serious adverse effects [78]. Consequently, further refinement and investigations into the efficacy and safety of MIP vaccines are imperative.

##### DAR-901

Like the *M. vaccae* vaccine and MIP vaccines, DAR-901 is derived from a heat-killed nontuberculous mycobacterium, specifically *M. kyogaense* sp. *nov*. Apart from serving as a preventive vaccine for boosting the Bacille Calmette-Guerin (BCG)-inoculated population, DAR-901 is also being evaluated as a potential therapeutic vaccine. Some studies have suggested that adjunctive treatment with DAR-901 can increase the conversion rates of sputum smears and sputum cultures as well as accelerate lesion resolution [228, 229]. This observed efficacy may stem from the ability of DAR-901 to increase the Th1-type immune response [230]. Nevertheless, variations in dosage, treatment duration, administration methods, and patient profiles, such as treatment-naïve, relapsed, or drug-resistant individuals, can significantly influence vaccine effectiveness [229]. Therefore, further exploration is imperative to elucidate the efficacy and potential applications of DAR-901 more comprehensively.

##### RUTI

Unlike the above 3 vaccines, RUTI is formulated from detoxified and fragmented *M. tuberculosis* cells. RUTI can induce Th1 immune responses against multiple *M. tuberculosis* antigens (e.g., ESAT-6, CFP-10, Ag85, and Hsp70), mediate a balanced immune response, enhance an effective cell-mediated response, and restrict excessive inflammation [231]. It has shown beneficial effects in preventing recurrence and treating latent TB infection (LTBI) in mice [232, 233]. The safety, tolerability, and immunogenicity of RUTI have also been assessed in a phase II clinical trial, where it demonstrated reasonable tolerability [84]. Furthermore, RUTI has been developed to increase its capacity for antigen presentation [234].

Advancing inactivated vaccines will require rigorous standardization of manufacturing protocols, particularly in strain selection, inactivation procedures, and morphological quality control. Such refinements are critical to achieving batch-to-batch consistency and reproducible immunogenicity. Subsequent clinical studies should systematically evaluate efficacy in key populations, including individuals with latent infection, HIV coinfection, or drug-resistant disease. Furthermore, strategic combinations of these vaccines with novel adjuvants or conventional chemotherapy may enhance immunotherapeutic outcomes and contribute to shortened, more effective TB treatment regimens.

#### Live attenuated vaccines

Live attenuated vaccines refer to low-virulence but highly immunogenic pathogens that can provide robust and long-lasting immunity. A typical example is BCG, an attenuated strain of *Mycobacterium Bovis* that has been used for more than 100 years [235]. BCG exerts its protective effects through various mechanisms, including improving immunity, promoting phagocytosis, regulating bidirectional immune responses, alleviating pathological damage, and establishing immune memory [217, 236]. Traditionally, BCG has been widely regarded as a preventive vaccine. However, given the aforementioned protective effects, the new role of BCG as a therapeutic vaccine has been explored [83].

To further enhance the efficacy, recombinant BCG (rBCG) vaccines have been developed that express *M. tuberculosis* antigens (e.g., Ag85B), human cytokines (e.g., IL-2), and molecules that activate innate immunity; and modify phagosome permeability [237]. Most rBCGs can enhance IFN-γ responses and *M. tuberculosis*-specific cell responses and thus offer robust protective effects in infected cells and experimental animals [238–240]. Notably, this type of vaccine has shown promising therapeutic potential for drug-resistant TB. Chiwala et al. [241] construct a recombinant drug-resistant BCG (RdrBCG) expressing Ag85B and Rv2628, and found it can reduce bacterial load and improve pathological damage in lungs of rifampin-resistant *M. tuberculosis*-infected mice. Despite their enormous potential, these candidates have not yet progressed into clinical trials, indicating a substantial amount of work to be done for their clinical application.

#### Adjuvanted subunit vaccines

Adjuvant subunit vaccines typically contain partial cellular components of *M. tuberculosis* and adjuvants that can increase immunogenicity [242]. Currently, only BCG polysaccharide nucleic acid (BCG-PSN) has been approved as an adjunctive therapy for TB patients in China [243]. Other vaccines are in various stages of clinical trials, with notable candidates such as H56:IC31 [86], ID93 + GLA-SE [244], and so on. H56:IC31 comprises three *M. tuberculosis* antigens (Ag85B, ESAT-6, and Rv2660c) coupled with an IC31 adjuvant. In animal models, H56:IC31 can induce multifunctional Th1 immunity, thereby protecting against *M. tuberculosis* [245, 246]. On the basis of these findings, its role as a therapeutic vaccine has been evaluated in a Phase I/II randomized trial involving pulmonary and extrapulmonary TB patients [86]. This trial confirmed the safety and immunogenicity of H56:IC31 in TB patients and highlighted its ability to significantly stimulate antigen-specific CD4^+^ T-cell proliferation. The mechanism of ID93 + GLA-SE is akin to that of H56:IC31, with ongoing clinical trials exploring its efficacy in treating TB patients [216].

Advancing adjuvant subunit vaccines will require the identification of novel immunodominant antigens and the rational design of adjuvant systems that elicit robust, multifunctional, and durable T-cell immunity. Future research should prioritize optimizing antigen-adjuvant synergy to enhance both the magnitude and quality of adaptive immune responses. Additionally, mechanistic studies elucidating the signaling pathways and memory formation induced by these vaccines are essential to guide their further development and clinical application.

#### Viral vector vaccines

Viral vector vaccines are created by incorporating *M. tuberculosis* antigen genes into a viral vector. By leveraging viral vectors, these vaccines simulate the process of *M. tuberculosis* invasion, eliciting robust and enduring immune responses [247]. Common viral vectors include modified poxviruses, adenoviruses, and lentiviruses. Ag85A and Ag85B are frequently utilized as antigens in vaccine development. An exemplary case is AERAS-402, a pioneer in clinical trials [248]. This vaccine employs a replication-deficient adenovirus as a vector and expresses 3 M*. tuberculosis* antigens, Ag85A, Ag85B, and TB10.4, known to trigger potent and sustained CD8^+^ and CD4^+^ T-cell responses [248]. Its safety and immunogenicity in active TB patients have been validated through a phase II clinical trial [248]. However, some researchers have noted that vaccine-induced T-cells fail to recognize *M. tuberculosis*-infected cells and control *M. tuberculosis* infection, thereby resulting in no discernible benefits during anti-TB treatment [249, 250]. Further exploration into the precise mechanisms underlying this shortfall in efficacy is essential to glean more insights for the development and evaluation of TB vaccines.

Various vaccines based on distinct viral vectors, such as chimpanzee adenovirus-based AdCh68Ag85A and modified vaccinia Ankara (MVA) virus-based MVATG18598, have exhibited potential in reducing bacterial loads and shortening treatment duration in animal models [251, 252]. Regrettably, their efficacy and safety in humans have not been validated through clinical trials.

Future development of viral vector-based TB vaccines should focus on optimizing vector design and antigen selection to enhance both CD4^+^ and CD8^+^ T-cell responses, particularly improving the recognition and targeting of *M. tuberculosis*-infected host cells. Key directions include engineering novel vectors with higher transduction efficiency and broader immunogenicity, as well as developing multivalent antigen formulations that elicit comprehensive and durable immunity. Further investigation into the mechanisms of immune evasion and memory T-cell formation will also be critical for the rational design of next-generation vaccines.

#### Nucleic acid vaccines

Nucleic acid vaccines represent a cutting-edge platform that has emerged over the past two decades, attracting significant attention owing to their notable benefits in terms of safety, swift design, and simple manufacturing processes [253]. These vaccines are constructed by incorporating DNA or mRNA sequences that encode the desired antigens into a vector. Upon administration, the nucleic acid molecule is transferred into the host cell, where it is translated and expressed. The expressed antigens can initiate antigen presentation and immune responses similar to those observed during pathogen infection, thereby providing protection to the host [254].

##### DNA vaccines

DNA vaccines have been a focus of earlier research efforts due to their favorable stability profile. Using bacterial plasmids as vectors, TB DNA vaccines can be developed by encoding genes encoding protective antigens from *M. tuberculosis* [255]. Several TB DNA vaccines have been developed, including rv2190c DNA, latency-associated DNA vaccines (especially rv2628 DNA), rv3407 DNA, BERopt DNA vaccines, Ag85A/B chimeric DNA and B21 DNA vaccines [171, 256–261]. These DNA vaccines exhibit strong immunogenicity and provide protective effects through various mechanisms. For example, they promote a shift from an inefficient immune response that mediates bacterial stasis to one that kills bacteria [262]. They can also restore the Th1/Th2 balance, the dysregulation of which is a mechanistic centerpiece of TB via enhancing the Th1 response and suppressing the excessive Th2 response [263–265]. In addition to their individual applications, combinations of DNA vaccines and other drugs have shown excellent therapeutic effects, resulting in a shortened treatment duration, prevention of TB reactivation, and so on [266–268]. However, it has also been reported that there is a risk of cross-reactivity and pathological autoimmunity due to the homology of DNA vaccine-related antigens to host proteins [269]. Therefore, the selection of target antigens should be performed carefully.

##### mRNA vaccines

In mRNA vaccines, exogenous mRNA encoding the antigen is typically delivered into cells utilizing lipid NPs, leading to expression of the antigen and the subsequent initiation of a robust immune response [270]. The mRNA vaccine platform offers many advantages, including versatility, rapid production, and the ability to elicit both cellular and humoral immune responses [270]. Nevertheless, mRNA vaccines have drawbacks. One significant limitation is their inherent instability, as mRNAs are prone to degradation by ubiquitous RNases, and these structures can be swiftly recognized as foreign by the innate immune system [271, 272]. In a seminal 2004 study, it was first demonstrated that RNA encoding the MPT83 antigen triggered protective immune responses against *M. tuberculosis* infection in mice [273]. Recently, advancements in immunoinformatics and related technologies have led to the emergence of various mRNA vaccine models, such as novel in silico mRNA vaccines [274] and MT. P495 [275]. In essence, while mRNA vaccines are still in their early stages, they hold immense promise for future development and application.

### Cell therapy

Cell therapy involves activating and expanding immune effector cells, either from patients themselves (autologous) or from a donor (allogeneic), in vitro [276]. These cells are then infused back into the patient to correct immune imbalances and enhance immune function, ultimately targeting and eliminating *M. tuberculosis* and infected cells [277]. Different types of cell therapy have varying effects, with T-cells playing a primary role, followed by mesenchymal stem cells (MSCs) (Fig. 2d).

#### T-cells

T-cells are a key component of cell therapy, and adoptive cell therapy (ACT) involves genetically modifying T-cells to express a chimeric antigen receptor or T-cell receptor (TCR) before reinfusing them into patients, especially in *M. tuberculosis*/HIV-coinfected patients and MDR-TB patients [278, 279]. For example, Zhou et al. [280] transfected CD8^+^ T-cells with a bispecific TCR that recognizes both *M. tuberculosis* and HIV-1 antigens, showing the potential for treating *M. tuberculosis*/HIV-1-coinfected patients. They also identified specific residues in the TCR responsible for T-cell responses (CDR3β: G115A, T116A, A117G, D114A, and S118A; CDR3α: E112A, Y115A, S113A, P114A, and S116A), providing insight for optimizing high-affinity bispecific TCRs [281, 282]. Liang et al. [87] used allogeneic Vγ9Vδ2 T-cells to treat MDR-TB patients and observed lung lesion repair, improvement in host immunity, and reduced *M. tuberculosis* burden. The safety of this type of cell therapy was also confirmed. Cytokine-induced killer (CIK) immunotherapy, which involves the infusion of autologous T-cells expanded in vitro, is another approach used to treat MDR-TB patients [90]. A clinical trial demonstrated that CIK therapy can promote sputum conversion to negative and improve clinical symptoms and imaging, as well as have a favorable safety [90]. Additionally, Chen et al. [283] validated the potential of Vγ2Vδ2 T-cells for treating MDR-TB or HIV-related TB using macaque models. These cells can differentiate into multifunctional effector cells and produce immune factors such as IFN-γ, perforin, and granulysin, effectively combating TB. Apart from the application scope and effectiveness of ACT, factors influencing its efficacy should also be considered, including the types of transfused T-cells and recipient characteristics such as age [284, 285].

Future progress in adoptive T-cell therapy for TB will depend on refining cell engineering techniques to improve antigen-specific targeting and functional persistence. Key research priorities include elucidating the mechanisms of T-cell trafficking, memory differentiation, and interactions with the immunosuppressive tubercular microenvironment. Additionally, optimizing manufacturing protocols and conducting rigorous preclinical validation are essential steps toward clinical application.

#### MSCs

MSCs, a heterogeneous cell population capable of differentiating into diverse lineages such as osteoblasts and neuronal cells, can interact with and modulate various immune cells, including the regulation of macrophage polarization. Khan et al. [286] reported that MSCs can work as phagocytic cells to control the replication of *M. tuberculosis* through autophagy and the release of NO. The transplantation of autologous MSCs has been shown to enhance the therapeutic efficacy for MDR or extensively drug-resistant (XDR)-PTB patients [88]. Another clinical trial verified the safety of MSCs as an adjunct therapy and reported common adverse events, including high cholesterol, nausea and lymphopenia [89]. While current evidence remains limited, the therapeutic potential of MSC-based therapy for tuberculosis warrants further exploration, particularly in optimizing delivery methods, elucidating detailed mechanisms of action, and validating efficacy in well-controlled clinical settings.

### ICIs

Immune checkpoints are surface proteins found on T-cells and other immune cells that serve as negative regulators of immune activation [287]. ICIs are a type of immunotherapy drug primarily used in antitumor treatments [288]. They work by alleviating the inhibition of antigen-presenting cells (APC)-mediated T-cell activation, restoring the antitumor immune response, and enhancing the clearance of tumor cells by T-cells [287]. Recently, dysfunction and exhaustion of T-cells has been observed in TB patients, suggesting the potential of ICIs in anti-TB treatment [289]. Among the various immune checkpoints, the PD-1/programmed cell death-ligand 1 (PD-L1) or PD-L2 pathway and Tim-3 signaling have emerged as promising targets in TB immunotherapy (Fig. 2e).

#### PD-1 and its ligands

PD-1, a type 1 transmembrane protein of the immunoglobulin superfamily, is expressed on various immune cells, particularly exhausted T-cells [290–292]. Its ligands, PD-L1 and PD-L2, are expressed on APCs, endothelial cells, epithelial cells and activated lymphocytes [293]. The PD-1/PD-L1 interaction inhibits CD3-mediated T-cell proliferation, and the PD-1/PD-L2 interaction dramatically inhibits TCR-mediated proliferation and cytokine production by CD4^+^ T-cells [294]. Overall, PD-1 and its ligands play a negative regulatory role in T-cell responses [294, 295]. This immunosuppression occurs through Src kinases-mediated phosphorylation of PD-1, which recruits SHP-2 to dephosphorylate key signaling molecules, ultimately leading to reduced cytokine production and T-cell proliferation [291, 296, 297]. The inhibition of distinct TCR signal transduction pathways, such as PKCθ and ERK activation, may also contribute to the negative immunoregulatory effect of PD-1 [293].

Studies have shown that the absence of PD-1 leads to an increased abundance of regulatory T-cells and MSCs and enhanced production of Th1, Th2 and Th17 cytokines but inhibits the proliferation of *M. tuberculosis* antigen-specific T-cells and macrophage autophagy. These alterations may result in increased susceptibility to *M. tuberculosis* infection [298, 299]. The absence of PD-1 expression is associated with increased inflammation and an increased *M. tuberculosis* load, indicating a protective role of PD-1 in host immunity against *M. tuberculosis*. However, Kamboj et al. [6] documented the negative effects of PD-1 on host anti-TB responses and demonstrated that PD-1 inhibition can restore the suppressed protective functions of polyfunctional T-cells. The combination of anti-TB chemotherapy and PD-1 inhibition has shown promising results in reducing the bacterial load in the lungs and spleen of infected mice, highlighting its therapeutic potential.

#### Tim-3

Tim-3 is a type 1 transmembrane protein expressed on various immune cells, including T-cells, monocytes, macrophages, and DCs [300]. Like PD-1, Tim-3 is considered a marker of T-cell exhaustion [301]. Tim-3 functions through interaction with its ligand galectin 9 (Gal9), which leads to the death of Th1 cells, induces macrophage activation, and promotes IL-1β secretion and pathogen clearance [302–304]. Tim-3 plays a direct role in adaptive immune responses against *M. tuberculosis* infection. When Tim-3-blocking antibodies are used, the production of IL-6, IFN-γ, and TNF-α increases, whereas the level of IL-10 decreases. This promotes macrophage activation and restricts bacterial growth [301].

ICIs represent a promising adjunctive strategy for TB treatment, particularly in drug-resistant and immunocompromised cases. Future research should focus on optimizing the timing and targeting of ICI interventions by elucidating the dynamics of checkpoint expression during *M. tuberculosis* infection. Combination therapies integrating PD-1 or Tim-3 blockade with conventional antibiotics may enhance bacterial clearance and shorten treatment duration. Rigorous clinical studies are needed to validate the safety, efficacy, and optimal dosing of ICIs in diverse TB patient populations.

## Challenges and future directions

Immunotherapy has emerged as a promising strategy against TB that addresses both *M. tuberculosis*-induced immune dysfunction and antigen-driven cell exhaustion. Current approaches, including immunoactive substances, chemical agents, vaccines, cell therapy and ICIs, have demonstrated superior anti-TB efficacy over existing treatments in both preclinical models and patient cohorts. Also, these immunotherapies have shown improved safety compared to standard chemotherapy regimens, which carry risks of severe adverse effects such as hepatic injury and neurologic problems [305]. However, larger scale clinical trials are needed to confirm their efficacy and safety, and to optimize administration methods prior to formal clinical application.

Although the advancement of single-cell techniques has allowed detailed characterization of immune cell subpopulation exhaustion in TB patients, the current mechanistic understanding remains limited. The prevailing hypothesis attributes exhaustion primarily to persistent *M. tuberculosis* antigen exposure, yet critical questions persist: both the drivers of such antigen persistence and alternative pathways inducing exhaustion remain poorly defined [28, 306]. A deeper exploration of the mechanisms underlying cell exhaustion could unveil potential targets to reinvigorate immune cell function and enhance host defense against *M. tuberculosis*. Additionally, there has been an excessive focus on T-cell exhaustion, while the attention given to exhaustion in macrophages, which are the primary *M. tuberculosis* host cells, and NK cells, which play important roles in antimicrobial defense, remains insufficient. These knowledge gaps are likely to impact the future development of immunotherapy strategies and their effectiveness.

Existing immunotherapies exhibit distinct advantages and limitations, as well as heterogeneous clinical maturity (Table 5). Cytokine-based therapies, such as IL-1 and IL-2, have progressed to clinical trials, while subunit and DNA vaccines remain exploratory. ACT harnesses the dynamic biological functions of engineered live cells, tailorable to specific therapeutic needs, to expand treatment options and enhance efficacy beyond conventional approaches [307]. However, TB-related ACT research lags behind that in other fields, with only a few candidates [e.g., chimeric antigen receptor regulatory T-cells (CAR-Tregs)] reaching phase I or II evaluation stages. Accelerating the development of ACT requires the establishment of comprehensive standards, such as determining the appropriate therapeutic cell lines for distinct TB states or *M. tuberculosis* strains, standardizing methods for modifying target cells, ensuring the survival and stability of target cells, and addressing potential rejection issues [308–310]. Beyond this, challenges persist in predicting the effects, as living cells exhibit microenvironment-dependent functions. For example, CAR-Tregs may exhibit instability and transform into inflammatory cells, leading to adverse effects [310]. While ICIs are predominantly studied in cancer patients, emerging evidence links their use to primary *M. tuberculosis* infection and latent TB reactivation [311–313]. This risk is further underscored by case reports documenting active TB onset in cancer patients receiving anti-PD-1 therapy [314–317]. Notably, Lee et al. [314] first reported *M. tuberculosis* reactivation in a Hodgkin’s lymphoma patient treated with the PD-1 inhibitor pembrolizumab, followed by a description of active TB in a nivolumab-treated lung cancer patient by Fujita et al. [315]. Similar cases have subsequently emerged across diverse ethnicities and solid tumor types [316, 317]. Retrospective cohort studies further confirmed elevated TB risk in patients receiving PD-1/PD-L1 inhibitors for specific cancers [318–320]. These findings do not appear to support the clinical application of ICI for TB. Of note, these phenomena are all observed among cancer patients receiving ICI therapy. A critical unresolved question is whether ICI dosing regimens designed to restore antitumor immunity inadvertently induce excessive T-cell activation and consequent inflammatory injury, thereby triggering TB reactivation. From a mechanistic perspective, PD-1 inhibition can promote TNF-α secretion, which exerts dual effects on *M. tuberculosis* infection control: moderate levels facilitate anti-*M. tuberculosis* defense through immune cell recruitment and granuloma stabilization, whereas excessive levels drive severe immunopathological injury [321, 322]. Therefore, dedicated clinical trials in TB patients are imperative to ascertain ICI efficacy and define TB-specific dosing regimens.

**Table 5:** Advantages and limitations of emerging immunotherapies for tuberculosis

Therapy strategies	Advantages	Limitations	Clinical trials stages
Immunoactive substances	Convenient administration; Diverse biological effects enabling comprehensive immune modulation	Short half-life; High treatment costs	Up to phase II
Chemical agents			
TLR-related chemicals	Rapid activation of immune responses	Risk of cytokine storm; Poor stability	NA^a^
Immunoxel	Convenient administration	Complex extraction and synthesis processes	Up to phase III
Nanotechnology	High targeting specificity; Low cytotoxicity; Excellent stability	High costs; Challenges in product quality standardization	NA
Vitamin D	Convenient administration; Low cost	Poor absorption; Variable effects at different doses	Unclear^b^
Vaccines	Simple administration; A few adverse effects	Poor sustainability; Restricted applicability (e.g., contraindicated in pregnancy)	Up to phase III
Cell therapy	High safety profile; Minimal or no side effects	High costs; Technically complex procedures (cell extraction, expansion, and reinfusion)	Unclear
ICIs	Durable therapeutic efficacy	Risk of immune-related adverse events (overactivation or autoimmunity); Potential reactivation of latent infections	NA

^a^No clinical trials initiated

^b^Clinical trials conducted (phase undisclosed)

*ICIs* immune checkpoint inhibitors, *NA* not applicable, *TLR* Toll-like receptor

In general, immunotherapy for TB has promising experimental efficacy as a primary or adjunctive therapy; however, its clinical translation remains limited. Key barriers include an incomplete understanding of *M. tuberculosis*-induced immune dysregulation and cell exhaustion mechanisms, hampering targeted therapeutic development. Moreover, existing studies have inadequately assessed the impact of subject demographics (e.g., age, sex, nutritional status) and disease type (e.g., active TB and LTBI) on therapeutic efficacy, complicating optimal patient stratification. For instance, as with conventional chemotherapy, the distinct immunological states in active TB and LTBI necessitate tailored therapeutic approaches involving differential dosing and duration [323, 324]. Nonetheless, few studies have compared the differences between these two types of patients regarding the same immunotherapy [56, 84]. Furthermore, the heterogeneous population of HIV/TB co-infection presents specific challenges. Despite the fact that certain immunotherapies exert excellent efficacy in this population, reported outcomes vary: for example, *M. vaccae* reportedly alleviated clinical symptoms and promoted sputum conversion in HIV-negative TB patients, yet failed to show efficacy in HIV-positive TB patients [69, 70]. Nevertheless, variations in study populations and dosing regimens between trials complicate the interpretation of these findings. Consequently, it remains difficult to determine whether distinct immunotherapy regimens are required for HIV-coinfected individuals. Critically, most of the recent research has focused primarily on efficacy while neglecting important factors such as administration methods, drug safety, treatment duration and dosage, causing it difficult to comprehensively assess the efficacy across different populations [55, 60, 88]. The advancement of TB immunotherapy necessitates a comprehensive approach that bridges fundamental research with customized clinical applications. Essential steps involve elucidating the immunopathological mechanisms underlying *M. tuberculosis* infection, formulating stratified immunotherapeutic strategies tailored to diverse disease states and specific patient demographics, and conducting thorough clinical evaluations that concurrently assess efficacy, safety, and intervention-related parameters. By undertaking these coordinated efforts, experimental immunotherapies can be effectively translated into broadly applicable clinical interventions for TB.

## Conclusions

In summary, this review examines the role of cell exhaustion in TB and the therapeutic potential of immunotherapies designed to reverse this immune dysfunction. We discuss key strategies, including immunoactive substances, chemical agents, vaccines, cell therapy, and ICIs, focusing on their mechanisms and current research progress. Mechanistic studies, clinical trials and multidisciplinary collaborations are essential for unraveling the complexities of *M. tuberculosis*-induced cell exhaustion and developing tailored immunotherapies for reducing the global burden of TB.

## Data Availability

Not applicable.

## Abbreviations

ACT: Adoptive cell therapy
Akt: Protein kinase B
albGM-CSF: Albumin-fused GM-CSF
AMP: Antimicrobial peptide
APCs: Antigen-presenting cells
ATT: Anti-TB therapy
ATRA: All-trans retinoic acid
BCG: Bacille Calmette-Guerin
BCG-PSN: BCG polysaccharide nucleic acid
CAR-Tregs: Chimeric antigen receptor regulatory T-cells
CIK: Cytokine-induced killer
CpG ODNs: CpG oligodeoxynucleotides
CTLA-4: Cytotoxic T lymphocyte antigen-4
DAT: Di-O-acyl-trehalose
DCs: Dendritic cells
DOTS: Directly observed treatment, short-course
ERK: Extracellular signal-regulated kinase
Gal9: Galectin 9
GM-CSF: Granulocyte–macrophage colony-stimulating factor
HDT: Host-directed therapy
HIV: Human immunodeficiency virus
ICIs: Immune checkpoint inhibitors
IFN-γ: Interferon-γ
IL-17: Interleukin-17
JAK: Janus kinase
LPS: Lipopolysaccharide
LTBI: Latent TB infection
MAPK: Mitogen-activated protein kinase
Mce3C: *M. tuberculosis* Effector mammalian cell entry 3C
MDR: Multidrug resistant
MDSCs: Myeloid-derived suppressor cells
MDT: Multidrug therapy
MHC: Major histocompatibility complex
MIP: M. indicus pranii
MMP-1: Matrix metallopeptidase-1
MSCs: Mesenchymal stem cells
*M. tuberculosis*: *Mycobacterium tuberculosis*
MVA: Modified vaccinia Ankara
NF-κ: Nuclear factor κ-
NK: Natural killer
NO: Nitric oxide
NPs: Nanoparticles
PAMPs: Pathogen-associated molecular patterns
PD-1: Programmed cell death protein-1
PD-L1: Programmed cell death-ligand 1
PDT: Photodynamic therapy
PGE2: Prostaglandin E2
PI3K: Phosphoinositide-3-kinase
PLGA: Poly(lactic-co-glycolic acid)
PRR: Pattern recognition receptor
PS: Phosphatidylserine
PTB: Pulmonary TB
rBCG: Recombinant BCG
RdrBCG: Recombinant drug-resistant BCG
RIPK1: Receptor-interacting protein kinase 1
STAT: Signal transducers and activators of transcription
TB: Tuberculosis
tBID: Truncated BH3-interacting domain death agonist
TCR: T-cell receptor
TF: Transfer factor
Th: T helper
Tim-3: T cell immunoglobulin mucin domain-containing protein-3
TLR: Toll-like receptor
TLR-4L: TLR-4 ligand
TNF-α: Tumor necrosis factor-α
TYK2: Tyrosine kinase 2
XDR: Extensively drug-resistant
